# Household stress moderates the association between caregiver metacognition and infant sustained attention

**DOI:** 10.1002/imhj.70038

**Published:** 2025-08-15

**Authors:** Ghada Amarieh, Line Caes, Alexandra Hendry, Sobanawartiny Wijeakumar

**Affiliations:** ^1^ School of Psychology University of Nottingham Nottingham UK; ^2^ Psychology, Faculty of Natural Sciences University of Stirling Stirling Scotland UK; ^3^ Department of Experimental Psychology University of Oxford Oxford UK

**Keywords:** caregivers, executive functions, infants, metacognition, SES, stress, sustained attention

## Abstract

Previous work has shown that caregiver executive functions (EFs) are robustly linked to EFs in children. However, existing evidence has used mixed methods approaches combining questionnaires and experimental tasks in older children. The current study used contextually similar questionnaires to examine whether caregiver EFs were linked to infant EFs, and whether household stress and socioeconomic status moderated these associations. Ninety‐one families living in the Midlands region of the United Kingdom participated in the study. Caregiver EFs were assessed using the behavior rating inventory of executive functions and infant EFs were assessed using the early executive functions questionnaire. Caregivers were also asked to provide information on household stress and socioeconomic status. Our findings showed that better caregiver metacognition was associated with better infant sustained attention, and this association was moderated by caregiver life stress. Our findings contribute to the understanding of early associations between caregiver and child EFs.

## 1 INTRODUCTION

During infancy, executive functions (EFs) such as working memory and inhibitory control undergo dynamic changes (Carlson, 2005; Diamond, 2013; Hendry et al., 2016). For example, at 4 months of age, infants can shift visual attention between different stimuli in their environment (Simmering, 2016; Swingler et al., 2018). By 6 months of age, they can detect and show preferential looking toward one changing item (Buss et al., 2018; Davidson et al., 2023; Simmering & Perone, 2013). By 10 months of age, they can maintain around two items in working memory (Ross‐Sheehy et al., 2003; Wijeakumar et al., 2023). By 10‐month‐old infants are also able to inhibit prepotent responses, gradually showing an improvement in inhibitory control between 18 and 24 months of age (Holmboe et al., 2021). Given these dynamic changes and longer‐term impacts, it is important to understand the role that contextual factors play on EF development,

Previous work has shown that cognition is heritable. For example, one study showed that primary caregiver and child EFs were correlated at 24 months of age, and that this association was stable through to 48 months (Cuevas et al., 2014). Similarly, another study found that both caregiver EFs and their caregiving practices contributed unique variance to EF development during toddlerhood (Ribner et al., 2022). During early years, caregivers who demonstrate better planning, self‐regulation, and attentional control, might be able to provide more consistent caregiving, maintain a structured environment, and engage in enriching interactions with their children (Diamond, 2013). There is mixed evidence for caregiver‐child EF associations in the first year of life. For example, previous work using experimental tasks has shown that only caregiver visual working memory behavior, but not inhibitory control is related to infant visual working memory behavior (Davidson et al., 2024; Theyer et al., 2024). In another study using mixed‐methods on the same cohort, caregiver behavioral regulation assessed using the behavior rating inventory of executive function (BRIEF‐A) questionnaire was associated with infant visual working memory behavior assessed using an experimental task (Amaireh et al., 2024). Given these mixed findings, it is important to examine whether such caregiver‐infant associations are present when EFs are assessed using contextually similar questionnaires.

Abundant research has shown that contextual factors such as household stress and socioeconomic status (SES) can impact caregiver and child EFs. For example, children from more affluent households display stronger inhibitory control and working memory skills compared to children from less affluent households (John, Kibbe et al., 2019). In rural settings, low SES has been linked to poor visual working memory behavior in infants and toddlers (Wijeakumar et al., 2019). Further, lower maternal education and household income was associated with weaker activation in the left frontal cortex during visual working memory processing. Other research has shown that heightened stress levels are linked to decreased EF abilities in caregivers (Bornstein et al., 2014; Romeo et al., 2022) and impaired inhibitory control and working memory in infants (Vrantsidis et al., 2020). More research is necessary to examine whether these contextual factors also moderate the association between caregiver and infant EFs.

In the current study, we investigated whether caregiver EFs were associated with infant EFs, and whether significant associations would be moderated by household stress and SES. We assessed EFs in caregivers using the BRIEF‐A and in their 9–10‐month‐old infants using the Early Executive Function Questionnaire (EEFQ). The EEFQ was developed for use with young children between the ages of 9 and 33 months (Hendry & Holmboe, 2021) and has been demonstrated to be sensitive to variance in EF up to 36 months (Hendry et al., 2022). Based on collective previous findings (Amaireh et al., 2024; Davidson et al., 2024; Theyer et al., 2024), we predicted that caregiver metacognition (related to working memory), emotional regulation (related to emotional control and shifting), and behavioral regulation (related to inhibition and self‐monitoring) would be linked to working memory in infants. Further, in line with previous work (Hackman et al., 2015; Lawson et al., 2018; Sarsour et al., 2011; John, Kibbe et al., 2019; Wijeakumar et al., 2019), we expected that both household SES and stress would moderate afore‐mentioned caregiver‐infant associations.

**Relevance and Key Findings**

- A model consisting of sustained attention, and persistence and regulation was observed in 9–10‐month‐old infants.
- Better caregiver metacognition was associated with better infant sustained attention.
- Life stress moderated the association between caregiver metacognition and infant sustained attention.

**Statement of relevance of the work for infant and early childhood mental health**

Our findings suggest that better metacognition in caregivers was associated with better sustained attention in 9‐to‐10‐month‐old infants. Importantly, this association was moderated by household stress.

## 2 MATERIALS AND METHODS

### 2.1 Participants

Families with infants aged between 9 and 10 months took part in the study through communication with relevant organizations and public engagement events. Interested families were screened to ensure they met the following inclusion criteria: (1) infants were born to full term and had caregivers with no history of illicit drug use or severe alcohol usage during pregnancy, (2) neither caregivers nor infants had been diagnosed with neurological or major psychiatric illnesses, (3) all infants had a normal or corrected‐to‐normal vision and (4) infants came from households where English was one of the primary languages spoken. To estimate sample size necessary for the current study, power calculations were carried out using G*Power for a linear multiple regression with fixed model, *R* ^2^ increase (effect size = .15, alpha = .05, power = .8, number of tested predictors = 3 and total number of predictors = 3). Based on this calculation, a total sample size of 77 was sufficient.

Ethical approval was granted by the School of Psychology Ethics Committee at the University of Nottingham (Approval Reference: F1415). A total of one‐hundred and twelve families living in the East Midlands region expressed interest in the project. Out of this sample, 4 families had infants who were not in the desired age‐range, 17 families withdrew from the project, three families had a history of color blindness and there was an experimenter‐related error with data from one family. Data was available from 87 families (infant age: 288 ± 15.4 days; 42 females, caregiver age: 33.47 ± 4.5 years; 86 females and one non‐binary). This sample also included a caregiver with twins. Of these families, 75.9% reported white British ethnicity, 2.3% reported another white ethnicity, 4.6% reported mixed ethnicity, 5.75 % reported Asian ethnicity, and 1.15% reported Arab ethnicity.

### 2.2 Caregiver EFs

The BRIEF‐A is a 72‐item questionnaire assessing adults’ self‐reported daily EF and self‐regulation (Roth et al., 2013). Caregivers were asked to answer the following question for each item: “During the past month, how often has each of the following behaviors been a problem?”. Caregivers responded with ‘Never’ (given a value of 0), ‘Sometimes’ (given a value of: (1) or ‘Often’ (given a value of 2). Items were categorized into nine EF subscales: inhibit, self‐monitor, plan/organize, shift, initiate, task monitor, emotional control, working memory and organization of materials. Raw scores were calculated by summing responses across relevant items for each scale. Thus, BRIEF‐A data was available from 86 caregivers.

We ran a confirmatory factor analysis (CFA) on list‐wise deleted data to confirm the three‐factor model from the original study by Roth et al. (2013). Communalities ranged from .33 to .73 in 8 items, and one item had a poor communality of .06. Factor scores were calculated and used in further analyses.

### 2.3 Infant EFs

The EEFQ is a comprehensive 31‐item questionnaire, consisting of 28 questions and 3 hands‐on activities, created to evaluate emerging EF skills in infants aged between 9 and 33 months (Hendry & Holmboe, 2021). The questionnaire covers items related to working memory, inhibition, cognitive flexibility, and regulation. Caregivers were asked to report how often in the last 2 weeks their child displayed behaviors using a 7‐item Likert response scale ranging from ‘Never’ (=1) to ‘Always’ (=7). Games were also incorporated to assist caregivers in interpreting the questionnaire items. These games were presented as optional. Since caregivers completed this questionnaire when their child was between 9 and 10 months of age, we only focused on items related to working memory and inhibition. We excluded items related to regulation and cognitive flexibility as previous work has shown that these functions do not emerge until 12–18 months of age (Kochanska et al., 1997; Shinya et al., 2022) and demonstrate floor effects in the first year of life (Hendry & Holmboe, 2021; Hendry et al., 2016). Also, we did not include data from the games as there was a variation in the tools used by caregivers to carry out the games in the lab and in homes. In total, 13 items were included in further analyses. Following coding instructions for the EEFQ (Hendry & Holmboe, 2021), two out of the 13 items were reverse‐coded (‘approach or reach for something that he/she has been repeatedly told not to touch such as electrical sockets or the oven’ and ‘seem to forget what they were doing, mid‐way through’). Data from 10 infants were not available for any EEFQ item. EEFQ data was available from a maximum of 77 infants. EEFQ data was not available for the pair of twins included in the study.

An EFA was run on list‐wise deleted data to extract factors of interest. Prior to initiating the EFA, we validated that the psychometric properties of the data. Specifically, we checked whether any item was highly correlated with any other item (>.8). This was not the case. The Kaiser—Meyer–Olkin measure of sampling adequacy was .55. We removed all items with measure of sampling accuracy < .5 and reran the analyses. Then, the Kaiser—Meyer–Olkin measure of sampling adequacy increased to .62, which is considered adequate (values ranging between .5 and 1.0 are indicative of appropriateness of the data). The Bartlett's Test of Sphericity was significant at the <.001 level (*X* ^2^ (36) = 100.9, *p* < .001). The overall Cronbach's alpha was .73, which is considered reliable. We carried out an EFA on the 9 remaining items using principal axis factoring and varimax rotation with a maximum of 25 iterations for convergence to extract the underlying factors. Note that we also confirmed that the same factorial structure was obtained when using oblimin rotation instead of varimax rotation. We also suppressed small coefficients using an absolute value below .32 (item loadings < .32 are viewed as undesirable for an EFA). Those factors with an eigen value of greater than 1 were chosen. If an item loaded on to two factors, then it was allocated to the factor where it had the highest loading. Communalities ranged from .28 to .64 for seven out of eight items, and one item had a low communality of .17.  Factor scores were calculated and used in further analyses. Note that a CFA based on Henry et al. (2021) was not conducted for a few reasons. First, there was a significant difference in the age range of the infants in both samples (9 to 30 months in Hendry et al., 2021 versus 9 to 10 months in the current sample). Second, for the afore‐mentioned reason, items related to cognitive flexibility and regulation were excluded in the current study. Third, both games related to working memory and inhibition were excluded due to inconsistency with the tools used in lab and home settings. The current study used only 13 out of the 31 items.

### 2.4 Household stress

The parenting stress index‐II is a 120‐item self‐report questionnaire regarding stressors. It consists of items making up the child stress domain, parent stress domain (caregiver‐related stress domain), and life stress domain(Abidin et al., 2013). We excluded the child stress domain as it contained items that were not applicable to infants as young as 9‐to‐10 months of age. The caregiver‐related stress domain referred to the assessment of caregiver characteristics that may be contributing to overall stress. It included 6 subscales covering competence, isolation, attachment, health‐related stressors, and so on. For this domain, a 5‐point Likert scale (1 representing ‘Absolutely disagree’ to 5 representing ‘Absolutely agree’) was used. The caregiver‐related stress score was calculated as the sum across all subscales.  The life stress domain referred to stress caused by factors outside the caregiver‐child relationship. It included 10 items such as divorce, marital reconciliation, marriage separation, and so on. For this domain, a dichotomous response scale of ‘yes’ or ‘no’ was used to assess each life stress item. Each item was given a particular score. The life stress score was calculated as the sum across all 10 items. Across both domains, higher scores indicated higher levels of stress experienced by the caregiver. Household stress data was available from 82 families (life stress: 9.5 ± 6.9, caregiver‐related stress: 88.3 ± 17.7).

### 2.5 Household SES

Caregivers were asked to respond to questions inquiring income and education level for themselves and their partners. For educational attainment, responses to level of education (e.g., secondary education etc.) were converted to years in education using government guidelines of education (https://www.gov.uk/further‐education‐courses). This calculation was done separately for caregivers and their partners (if available) and then summed together to generate household educational attainment in years.

Primary caregivers were asked to provide their income after tax. They were also asked to provide either income after tax or income bracket for their partner. If income bracket was provided (e.g., 10K–20K), then the mid‐way point was chosen as the income (e.g., 15K if 10K–20K bracket was chosen). Caregiver and partner incomes were summed to calculated household income. Household SES data was available for a total of 74 families (income: 60,232 ± 19,580, educational attainment: 29.5 ± 5).

### 2.6 Procedure

After screening, families were invited to visit the research facility. Caregivers gave consent for themselves and their infants. The project had multiple assessments, and caregivers attended closely spaced visits in the first year. The BRIEF‐A questionnaire was completed in the lab when the infants were between 6 and 10 months. The EEFQ was completed in the home when the infant was between 9 and 10 months, using a Qualtrics link that was emailed to the families. Families were renumerated for each visit to the lab.

### 2.7 Outlier removal

Prior to statistical analyses, caregiver EF factor scores, infant EF factor scores, household income, household educational attainment, caregiver‐related stress, and life stress were examined for outliers. A data point was considered an outlier and removed if it fell outside of ±3 SDs from the mean. Life stress and household income both had two outliers each, which were removed before moderation analyses.

### 2.8 Regression and moderation analyses

Regression models were run using RStudio (RStudio Team, 2020) to examine whether caregiver EFs were associated with infant EFs. In all models, the caregiver EF factor was the predictor variable, and infant EF factor was the outcome variable.

Next, we examined whether significant associations between caregiver and infant EFs were moderated by household income, educational attainment, caregiving stress or life stress. Separate models were run to examine the unique contributions of each moderator without the potential confounding effects of including multiple moderators simultaneously. Conditions for moderation analysis were set to probe interactions of *p* < .10 to test for simple slopes within the model. To illustrate the moderation in the graphs, the moderator variables were split into three groups: low levels of the moderator (i.e., −1SD of the mean), average levels (i.e., mean value), and high levels of the moderator (i.e., +1SD of the mean). As there is abundant research on the influence of SES and stress variables on infant abilities, theoretical justification outweighed the need for statistical corrections for the number of moderation models.

## 3 RESULTS

### 3.1 Factor structure for caregiver EFs

The CFA demonstrated that Roth et al. (2013)’s three‐factor model was a good fit to the current data. This model consisted of emotional regulation (shift and emotional control), behavioral regulation (inhibit and self‐monitor) and metacognition (plan/organize, organization of materials, working memory, task monitor, initiate). The model indices suggested good fit. The Chi‐Square value for the default model was low and not significant (*X* ^2^ (24) = 27, p = .32). The Comparative Fit Index and Tucker‐Lewis Fit Index were .99 and .99, respectively. Additionally, the Root Mean Square Error of Approximation (RMSEA) was .04 with 90% confidence interval of .00 to .1. Across all factors, higher scores are indicative of poorer abilities (Table 1).

**TABLE 1:** CFA factors from the BRIEF‐A based on the Roth et al. (2013) three‐factor model.

No.	BRIEF‐A subscales	CFA factors
1.	Shift	Emotional regulation
2.	Emotional control	
3.	Initiate	Metacognition
4.	Working Memory	
5.	Plan/organize	
6.	Task monitor	
7.	Organization of materials	
8.	Inhibit	Behavioral regulation
9.	Self‐monitor	

### 3.2 Factor structure for infant EFs

After examining the scree plot and prioritizing eigen values of >1, the EFA showed that a three‐factor model best fit our data and accounted for 62% of the variance (Table 2). The first factor consisted of four items and was conceptualized as “sustained attention” because contributing items referred to infants’ ability to remain focused without getting distracted. The second factor consisted of four items and was conceptualized as “persistence and regulation” because it referred to how infants pursued an action or a goal until they were successful. The third factor consisted of only one item, so a factor score was not computed, and this item was excluded from further analyses. Across the included factors, higher factor scores are indicative of better abilities.

**TABLE 2:** The included two EFA factors from the EEFQ.

No.	EEFQ items	EFA factors
1	Seem to forget what they were doing, mid‐way through	Sustained attention
2	Follow a simple instruction for a task that s/he was interested in (e.g., getting a nearby toy), without getting distracted	
3	Continue with what they had been doing after having been interrupted by a minor distraction (such as having clothing/socks adjusted)	
4	Repeat or copy something they had just been shown how to do (e.g., work a tricky toy)	
5	Stop reaching completely for something when you said “no/don't touch” or similar	Persistence and regulation
6	Go after something they wanted (e.g., your phone or the remote control for the TV) even after you had just hidden it from view	
7	Repeat a new skill or action until he or she could do it (e.g., grabbing a toy that's almost out of reach)	
8	Re‐try an action slowly and carefully (for example to get a shape in a shape sorter, or to catch a dangling toy)	

### 3.3 Association between caregiver EFs and infant EFs

**FIGURE 1:**
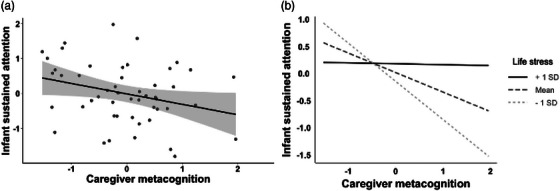
(a) Better caregiver metacognition (lower scores) was associated with better infant sustained attention (higher scores). (b) Caregiver life stress moderated the association between caregiver metacognition and infant sustained attention.

Six regression models were run to examine the association between each of the three caregiver EF factors and two infant EF factors (e.g., model 1: caregiver metacognition and infant sustained attention, model 2: caregiver metacognition and infant persistence and regulation…). There was a significant association between caregiver metacognition and infant sustained attention (*t* = −2.12, *p* = .039, *R* ^2 ^= .09, ß = ‐.30). Here, better caregiver metacognition (lower scores) was associated with better infant sustained attention (higher scores)—see Figure 1a. These effects remained significant even after controlling for infant age in days.

### 3.4 Moderating influences

Four moderation models were run to examine the effect of each of the four moderators (household educational attainment, household income, caregiver‐related stress, and life stress) on the afore‐mentioned significant caregiver–infant EF association. Our findings revealed that only caregiver life stress significantly moderated the association between caregiver metacognition, and infant sustained attention. Specifically, there was a significant interaction between caregiver life stress and caregiver metacognition (*b* = .05, SE = .02, *t* = 2.3, *p* = .027). When examined further, the simple slope of caregiver metacognition on infant sustained attention was significant for households with fewer (*b* = −.70, SE = .22, *t* = −3.16, *p* < .01) and mean (*b* = −.36, SE = .14, *t* = −2.55, *p* = .01) reported life stressors—see Figure 1b.

## 4 DISCUSSION

The overarching aim of the study was to examine whether caregiver EFs was associated with infant EF factors, and if these associations were moderated by household stress and SES. We predicted that caregiver metacognition, emotional regulation, and behavioral regulation would be associated with factors related to working memory in infants. Further, we expected that both SES and household stress would moderate significant caregiver‐infant associations. In partial support, we found that better caregiver metacognition was associated with better infant sustained attention. In caregivers, the metacognition factor was made up of items related to working memory. Similarly, in infants, the sustained attention factor is closely linked to working memory, that is, to demonstrate better sustained attention, object and/or goal representations must be robustly held in working memory and distractors must be suppressed. In addition, the four items making up this factor were categorized as related to working memory in Hendry et al. (2021). This working memory‐related association between caregivers and infants aligns with previous work showing that caregiver visual working memory was positively linked to infant visual working memory when assessed using age‐appropriate preferential looking tasks in the same families as the current study(Theyer et al., 2024). Collectively, these findings highlight the heritable nature of working memory in caregivers and children, as early as infancy—using both experimental tasks and questionnaire‐based methods. These heritability‐based effects could also be linked to interaction‐based effects. For example, caregivers who have better metacognitive abilities might be better able to initiate, guide, and sustain their infants’ attention during interactions—thus, producing robust caregiver‐infant associations, compared to caregivers with poor metacognitive abilities.

Only caregiver‐reported life stress moderated the association between caregiver metacognition and infant sustained attention—with this effect being stronger in households with fewer reported stressors. It is possible that in households with fewer life stressors, caregivers with better metacognitive abilities might be less occupied or distracted and are able to observe their infants’ abilities (and report on them) and guide their cognitive resources—thus, producing more robust associations in working memory between these caregivers and their infants. In general, this finding also aligns with previous work showing that a higher frequency of stressful life events is linked to poorer visual working memory processing in preschool children(McKay et al., 2021).

Despite abundant previous evidence linking SES to caregiver and child EFs(Hoff, 2003; Lawson et al., 2018; Petrill et al., 2004; St. John, Finch et al., 2019; Wijeakumar et al., 2019), it did not moderate caregiver‐infant EF associations. It is possible that, in most households, the impact of SES on caregiver and child cognition begins to emerge only during toddlerhood and preschool periods when there is a greater need and dependence on household enrichment, and caregiver awareness, time and involvement. Future work should aim to investigate whether and how some, compared to other contextual factors are relevant for specific developmental time‐points. A further consideration was that caregiver behavioral regulation was not associated with any infant factors. This result contradicts recent work showing a link between caregiver behavioral regulation assessed using the BRIEF‐A and visual working memory in infants assessed using a preferential looking task (Amaireh et al., 2024). It is possible that caregiver‐infant associations are not observable when EFs are assessed using mixed methods. For example, some questionnaire items and/or caregiver responses to those items based on observations of their infants’ behaviors might not tap into the same mechanisms as those directly engaged by infants during experimental tasks. Further, we acknowledge that there may be biases that may arise from the use of questionnaires as caregivers may have similarly reported on themselves and their infants.

Lastly but importantly, while the current study revealed interesting caregiver‐infant EF associations and moderation influences, future work aiming to replicate this work or use the same methods on similar cohorts should increase the sample size to improve model stability for running EFAs and moderation analyses

The current study aimed to understand whether caregiver EFs were associated with infant EFs and if these associations were moderated by SES and household stress. We found that better caregiver metacognition was associated with better infant sustained attention. Importantly, life stress moderated this association, with this effect being stronger in households with fewer reported stressors.

## CONFLICT OF INTEREST STATEMENT

No potential conflict of interest is reported by the authors.

## Data Availability

Data will be made available upon reasonable request.
